# Do Remittances Enhance Elderly Adults’ Healthy Social and Physical Functioning? A Cross-Sectional Study in Nigeria

**DOI:** 10.3390/ijerph19041968

**Published:** 2022-02-10

**Authors:** Nnaelue Godfrey Ojijieme, Xinzhu Qi, Chin-Man Chui

**Affiliations:** 1 School of Public Policy and Administration, Xi’an Jiaotong University, Xi’an 710049, China; nnaelue@yahoo.com (N.G.O.); qixinzhu@126.com (X.Q.); 2 School of Business, Macau University of Science and Technology, Taipa, Macau 999078, China

**Keywords:** healthy aging, social functioning, physical functioning, remittances, adjusted predicted probabilities, predictive margins, marginsplot

## Abstract

Existing research demonstrates that the effect of remittances on different health outcomes of elderly adults in low-income countries with inadequate social security is inconclusive. The present study set out to fill this gap by examining the effects of receiving remittances on the healthy social and physical functioning of Nigeria’s elderly adults. We also investigate the nonlinear association between remittances and social and physical functioning to identify the minimum amount required to engender healthy social and physical functioning in Nigerian elderly adults. This study utilized data from the 2018/2019 Nigerian Living Standard Survey (NLSS), which included 55,350 young people aged 12–44 (control group) and 18,937 elderly adults aged 45 and above (treatment group). We addressed our objectives using logistic regression-adjusted predicted probabilities and predictive margins. The results reveal that remittance recipients have higher healthy social functioning probabilities than non-recipients. We also found that the influence that remittances have on social functioning depends on the amount of the remittances received. Quantitatively, receiving more than NGN 1,200,000 in remittances ensures increased social functioning probabilities. Given the disparity between the average remittance amount of NGN 54,306.92 received by elderly adults and the NGN 1,200,000 threshold associated with healthy social functioning, bridging this gap is paramount for promoting social functioning among Nigerian elderly adults. We also discussed policy implications for fostering the healthy aging of the population in the interim.

## 1. Introduction

Population aging is an essential demographic phenomenon of the 21st century that is fueled by the expansion of elderly adults around the world. The World Health Organization (WHO) predicts the population of elderly adult to double and outgrow the population of children (aged five and below) between 2015 and 2050 [1]. Similar to other low-income countries, the United Nations (UN) has projected Nigeria’s elderly adults’ population to grow by 200% between 2016 and 2050 [2]. The transition to an aging society requires efficient socioeconomic transformation to maintain the healthy aging of the population [3,4,5]. Healthy aging, which promotes aging with good social, physical, and mental functioning [6], is a significant challenge in low-income countries such as Nigeria due to inadequate social security and financial infrastructure to address issues related to healthy elderly aging [7]. In the interim, these challenges inform global and local strategies for sustainable socioeconomic-augmenting financing to optimize the healthy aging of the increasing elderly population.

Social and physical functioning, which captures the ability to engage in social activities (such as participation in the labor force) and to perform daily physical routines, respectively, are two age-related healthy aging measures eluding Nigerian elderly adults. Based on the life-course aging theory, environmental and socioeconomic factors reduce the ability of elderly adults to maintain these functions as they age [8,9,10,11]. Hence, in low-income countries such as Nigeria, socioeconomic factors such as inadequate financial and family support are the leading inhibitors of healthy aging [12,13,14,15]. Consequently, most Nigerian elderly adults have insufficient finances to seek medical redress, though 60–80% have multimorbidity challenges requiring regular medical attention, posing a substantial challenge to healthy aging [16]. Therefore, non-conventional financing sources such as remittances are growing more relevant as an alternative source of health-financing income [17,18,19].

Remittances are essential sources of income in Nigeria, as they comprise up to 40% of the annual income of elderly adults required in order for them to live above the national poverty line (The poverty line is defined as an annual income of NGN137,430, while the average amount of remittances received by elderly adults in Nigeria is NGN 54,306.92 [20]). Remittance inflow also compares to 83% of Nigeria’s national budget [21]. Furthermore, researchers have recognized and discussed the importance of remittances for improving the social, physical, and mental health of elderly adults [22,23]. However, though remittances encompass financial supports from family and other sources, limited studies have attempted to quantify this “support,” as they have focused on perceived support. Hence, given people’s subjective perception of “support,” we attempt to eliminate this bias by quantifying this support using the monetary and support received in-kind from families and other groups—remittances.

Therefore, utilizing the Nigerian 2018/2019 Living Standard Survey data released in 2021, this study attempts to bridge this gap by employing the logistic regression method to estimate the association between remittances and healthy aging using two essential measures—social and physical functioning. We also utilize the predictive margins tool to predict the social and physical functioning probabilities for recipients and non-recipients of remittances using elderly adults as the treatment group and the young population as the control group. 

### Literature Review

Healthy aging is a preferred concept because, unlike active aging, it allows for differentials in what constitutes good health outcomes [24]. For instance, elderly adults could be aging healthily even though they are less physically or socially capable than children; hence, comparing people of the same demography provides better health measures. The Life-course aging theory is the basis of the argument that the health status of elderly adults reflects their accumulated environmental, physical, and mental activities from childhood [9]. Several indicators such as quality of life, physical, cognitive, physiological, and social functioning measure healthy aging in the literature [6,10,11,25].

Regarding healthy aging determinants, sociodemographic factors dominate contemporary literature in low-income countries. For instance, since elderly adults have more life stressors, Kernisian [26] argued that age is a credible determinant of healthy aging. Additionally, Zhong et al. [27] found that regular walking and social networking activities are credible healthy aging influencers. Higher education [28], shorter heights [29], and behavioral factors such as diet, smoking, and alcohol consumption are other viable determinants [30,31,32]. Interestingly, evidence suggests that crucial healthy aging determinants vary between countries. For example, in medium-to-high-income countries, Campos et al. [4] found that gender-related quality of life, income, and community participation are the determinants of healthy aging in Brazil, and Cahyaningtyas et al. [6] introduced nutritional intake, religious beliefs, and perceived financial position as influential factors in the United States (US). However, in low-income countries such as Cameroon and Zambia, socioeconomic factors such as income and income accessibility, employment status, education, and social networks dominate literary discussions [5,10].

Previously, the United Nations also emphasized socioeconomic inadequacies as a significant inhibitor of healthy aging in low-income countries, with only 17% of the elderly population in Sub-Saharan Africa’s (SSA) receiving a pension or other social security support, painting a picture of gloomy prospects for the elderly population in SSA countries [2]. Some scholars have also identified effete social security and broken family support systems as significant problems that are faced by elderly adults in low-income countries [7,33], and this situation is worse in Nigeria due to high unemployment [34], chronic poverty [35], and the government’s inability to adequately fulfill its salary and pension payment responsibilities [36].

**Figure 1:**
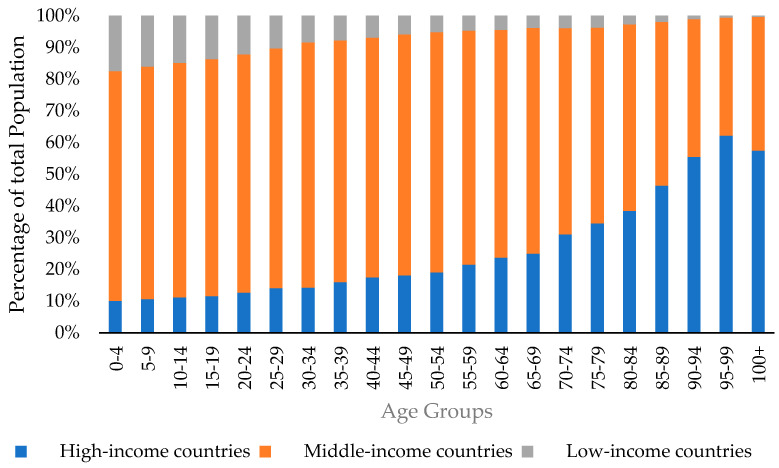
The global distribution of the population by age groups.

This possibly explains the historical low life expectancy in low-income countries, as observed in Figure 1 below. The number of people surviving through adulthood shrinks alarmingly relative to high-and-middle-income countries. Specifically, elderly adults (aged 45 and above) constitute 44% of the population in high-income countries, but only 14% of the population in low-income countries and 12% in Nigeria [37,38]. The earlier socioeconomic inadequacies that were discussed earlier render Nigerian elderly adults financially vulnerable in the face of substantial medical and other needs [16]. Hence, there is a need for a national strategy to promote a sustainable alternative source of income in the short term. The authors of [17,18,19] have argued that remittances can be this alternative source of income.

Remittances have far-reaching impacts in reducing poverty and inequality and in fostering socioeconomic development [39,40]. It smooths out consumption and household expenditures on a micro level since households allocate this additional income for other critical alternative uses such as health [41,42]. Remittances are essential sources of income in Nigeria, as highlighted earlier. Researchers have also found that remittances increase health knowledge and improve access to healthcare [43,44]. Additionally, [45,46] found that remittances improved life expectancy and reduced infant mortality. Conversely, some researchers argued that remittances could not adequately compensate for loneliness, poor mental health, and bodily wear and tear associated with labor participation by elderly adults left behind due to migration [47,48,49]. These findings are not nationally representative and did not examine whether the effect of remittances depended on the amount of the remittances received. In fact, studies highlighting the nonlinear relationship between remittances and other indicators suggest that healthy aging could also depend on the amount of remittances received [50,51,52]. For instance, [18] found that receiving more than INR 35,000 improved the self-reported health status of left-behind Indian wives. Hence, ignoring this nonlinear association possibility would deny us valuable information about the true nature of the relationship between remittances and healthy aging in Nigeria.

Existing research demonstrates that the effect of remittances on different health outcomes in elderly people in low-income countries with inadequate social security is inconclusive. Additionally, current studies from low-income countries have dwelt on perceived support, leaving a gap for our study to quantify this support. The present study addressed this gap by examining the effects of receiving remittances on two important healthy aging indicators—social and physical functioning. Addressing this gap is relevant in the context of low-income countries with effete social security systems but have remittances playing significant roles in the economy and lives of elderly adults. Ultimately, we will ascertain the remittances required to engender healthy aging in Nigerian elderly adults.

## 2. Materials and Methods

This study utilized data from the Nigerian 2018/2019 Living Standard Survey (NLSS), which was conducted by the National Bureau of Statistics (NBS) and was made public in 2021. The survey captured information on Nigeria’s socioeconomic and demographic characteristics. It covered all eligible households, excluding prisons, hospitals, military barracks, school dormitories, and Borno State violent conflict areas. The survey adopted a stratified four-stage cluster design—the States, Enumerations Areas (EAs), Households, and Individuals. The NLSS contains 200 EAs from each of the 36 states and the Federal Capital Territory (FCT) (comprising 20 replicates of 10 sample EAs) that are drawn systematically from all the Local Government Areas in Nigeria. Next, 60 of 200 EAs were selected using systematic random sampling from 36 states and the Federal Capital Territory. Finally, ten households were chosen from these 60 EAs, comprising 22,200 households and 116,320 individuals nationally. Given that there are no responses on the marital status of respondents less than 12, and four older respondents not answering questions about whether they were receiving remittances, we excluded these missing data to capture a weighted sample of only 74,287 individuals aged 12 years and above.

### 2.1. Outcome Variables

This study adopts two health aging measures from the survey—the Social Functioning (SF) and Physical Functioning (PF) of elderly adults. They are two of the most relevant healthy aging measures in the literature [53]. Additionally, insufficient data from the survey limited our choice of healthy aging measures. Furthermore, we could not combine both measures due to their Cronbach’s Alpha of 0.471. SF is a valid indicator capturing one’s ability to engage in socioeconomic activities. Hence, in this study, SF captured participants that responded 1 (yes) or 2 (no) to the questions on whether they have worked (1) a wage job, (2) on their own agricultural facilities, (3) in their own nonfarm enterprises, or (4) as trainees or apprentices anywhere in the past seven days. In the current study, “yes” responses to any question are coded as 1—high functioning (64%), while “no” responses to all the questions received a score of 0—low functioning (36%).

PF, also referred to as Activity of Daily Living (ADL), is a subjective health measure where participants self-assess their health. In the Nigerian Living Standard Survey (NLSS), the participants responded to PF questions that included whether they had difficulties (1) seeing, even if wearing glasses; (2) hearing, even if wearing a hearing aid; (3) walking or climbing steps; (4) remembering things and concentrating; (5) bathing, dressing, feeding, using the toilet, and so on; (6) and communicating. They ranked their difficulty levels from 1 (no difficulty) to 4 (cannot). In the current study, respondents experiencing “no difficulties” were coded as 1—high functioning (90%), while respondents responding “yes, some difficulties” to “cannot” were coded as 0—low functioning (10%).

### 2.2. Independent Variables

Two aspects of remittances make up this study. One facet of remittances is a binary variable that indicates whether remittances of monetary or in-kind are (received remittances = 1) or not (received remittances = 0) within or outside of Nigeria. This was determined to enable the interaction with the “population group” variable and to show the effect that receiving remittances has on the social and physical functioning of older adults relative to the younger population. Next, for our assessment of a nonlinear relationship between remittances and the health variables and the loss of information and other statistical troubles associated with dichotomizing a continuous variable, we also accounted for the total remittances received by elderly adults [54,55,56]. We calculated remittances by summing the total monetary value of all cash and in-kind assistance within and outside Nigeria. While some of the participants received all four forms of assistance (cash assistance from Nigeria, cash assistance from abroad, in-kind assistance from Nigeria, and in-kind assistance from abroad), others received none, one, two, or three forms of assistance. We summed all receipts by all of the participants who received at least one form of assistance (30%) and those that received none (70%).

Next, to ascertain the effect of remittances, we stratified the age groups such that the young—12 to 44 years (75%)—population represented the control group, while middle-aged—45 to 65 years (19%)—and the elderly—66 to 120 years (6%)—represent the treatment group. In our research, several elderly studies influenced our decision to classify the middle-aged as an elderly group [14,57]. In a Nigerian study, Chukwuorji et al. emphasized that a low life expectancy of 54 years makes implementing the elderly benchmark of 60 years from other climes unreasonable. Hence, given that not many Nigerians survive past 54, an elderly benchmark of 44 is appropriate for the Nigerian context.

### 2.3. Other Demographic Variables

Following recent works [14,58], we adopted the following sets of covariates associated with the health of elderly adults. We coded gender as 1—males and 2—females. Marital status comprised seven categories: (1) married (monogamous), (2) married (polygamous), (3) informal/loose union, (4) divorced, (5) separated, (6) widowed, and (7) never married. We coded 4, 6, and 7 as unmarried in this study and 1, 2, 3, and 5 as married. Because English is a second language that is formally learned in schools, being proficient in its use is a massive indicator of literacy. Hence, we coded literacy (1—illiterate and 2—literate) as whether respondents were able to read and write in English. We coded residential areas as 1—rural and 2—urban. Finally, the “geopolitical zone” indicates the six geopolitical zones of Nigeria, which represent different cultural and socioeconomic characteristics—the northeast, northwest, north-central, southeast, south-south, and southwest, which were coded 1–6, respectively.

### 2.4. Statistical Model

We employed logistic regression to investigate the relationship between the outcome variables for healthy aging (The Social Functioning (SF) and Physical Functioning (PF)) and the independent variables (age groups, remittance status, and remittances received) after controlling for other sociodemographic variables discussed in the previous subsections. The following model was used for basic estimation:

log[*prob*(*Y_i_* = 1)/*prob*(*Y_i_* = 0)] = *β*_0_ + *β*_1_*Age groups* + *β*_2_*remittance status* + *β*_3_*Age*
*group* * *remittance status* + *β*_4_*remittance amount* + *β*_5_
*remittance amount*^2^ + Σ*β_i_*
*sociodemographic variables_i_*

where *Y_i_ = SF or PF,* 1 = high functioning, and 0 = low functioning. The left-hand side of the logistic regression model measures the log of the odds ratio. Increasing the independent variable or demographic variable by 1 unit will result in an increase in the *β_i_* units in terms of the log of the odds ratio. Now, if the log of odds ratio increases by *β_i_* units, that means *prob*(*Y* = 1)/*prob*(*Y* = 0) will increase by *exp*(*β_i_*).

### 2.5. Statistical Analysis

First, we present the descriptive statistics to describe the characteristics of the sample utilized in our study. Second, we survey-set our data to guarantee that the estimates yielded the appropriate standard errors. Utilizing STATA 16 [59], we generated the Adjusted Odd Ratios (AORs) for our models with logistic regression analysis. Finally, we employed the tools “margins” and “marginsplot” to address our research objectives. The margins command in STATA generates the adjusted predicted probabilities of attaining social and physical functioning for the treatment and control groups and is conditional on the average values of all of the variables in the model. This tool helps to highlight the effect of remittances by clarifying the within-group differences in the outcomes between the treatment and controls in our three population groups. It also predicts the marginal effects of receiving an increasing remittance amount on the probabilities of SF and PF. In model one, we assessed the nature of the relationship between remittances and SF and the effect of receiving remittances on the SF odds for the treatment group. We repeated the same process for the second outcome variable in model two—PF.

## 3. Results

### 3.1. Sample Characteristics

Table 1 notes that 60% of the population of elderly adults (average of middle-aged and the elderly) is not literate. This reality has adverse implications for obtaining high-salary jobs, pension, and insurance benefits, ultimately limiting the ability of this population to attain healthy social and physical functioning. Elderly adults constitute 26.1% of the population, indicating that Nigeria is predominantly youthful. Additionally, with 45% of elderly adults receiving remittances, we see that remittances are an integral source of income for Nigerian elderly adults. The importance of remittances is not just a Nigerian phenomenon, as remittances are common in low-income countries relative to high-income countries [60]. Next, most elderly adults are married (77%) and reside in rural areas (70%). Finally, the population of elderly adults is evenly distributed in terms of gender.

### 3.2. Regression Result

#### 3.2.1. Association between Remittances, Social and Physical Functioning

The logistic regression in Table 2 shows that remittances are significantly associated with the SF of elderly adults (AOR = 1.000; CI = 1.000–1.000; *p* < 0.01) and PF (AOR = 1.000; CI = 1.000–1.000; *p* < 0.1). However, the non-linear term of remittances is only significantly associated with the SF of elderly adults (AOR = 1.000; CI = 1.000–1.000; *p* < 0.05). (Although minute, the significance of the coefficients of the remittances (−1.92 × 10^−6^) and remittances^2^ (7.51 × 10^−13^) informed the non-linearity argument).

#### 3.2.2. The Effect of Receiving Remittances on Social and Physical Functioning

**Figure 2:**
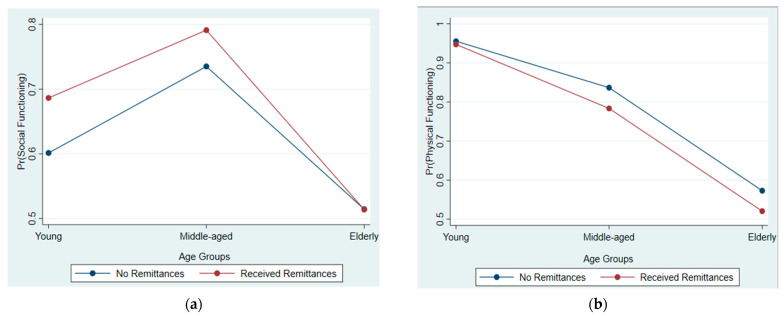
(**a**) Adjusted predicted probabilities of SF by population groups and remittance receipt status. (**b**) Adjusted predicted probabilities of PF by population groups and remittance receipt status.

Additionally, we observed that despite receiving remittances, the elderly has lower SF odds (AOR = 0.655; CI = 0.532–0.807; *p* < 0.01), while the middle-aged population has lower PF odds (AOR = 0.840; CI = 0.717–0.985; *p* < 0.05) relative to the younger population not receiving remittances. Nonetheless, the adjusted predicted probabilities and marginsplot of our logistic model reveal that the average young and middle-aged recipient of remittances has higher SF probabilities than non-recipients of remittances. The probability is about even for the elderly, regardless of whether they are receiving remittances or not (see Table 3 and Figure 2a). In contrast, the average recipients of remittances from each group have lower PF probabilities than non-recipients of remittances (see Table 4 and Figure 2b).

**Figure 3:**
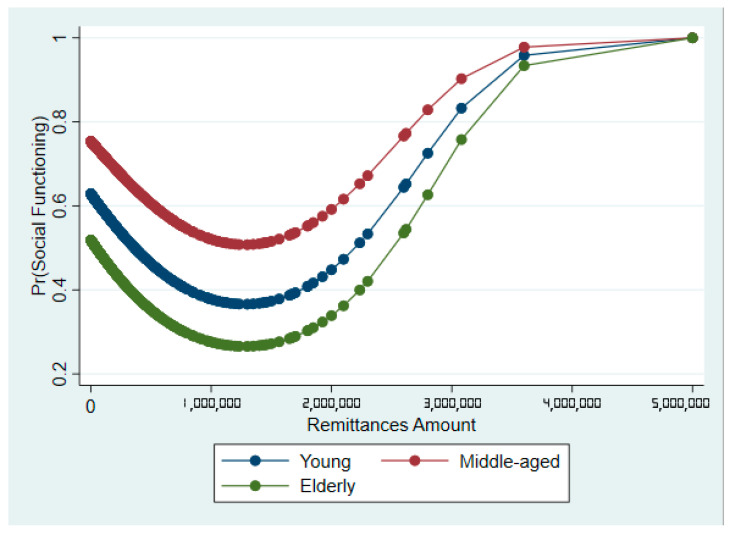
Predictive margins of SF by population groups and remittance amount.

Finally, by predicting each population group’s SF probabilities for additional remittances received, the predictive margins (captured by the marginsplot in Figure 3) show the nonlinear relationship between SF and remittances. It reveals that remittances are only positively associated with SF when they reach a certain threshold. First, the predicted SF probability falls for all groups receiving yearly remittances less than NGN 1,200,000. After this threshold, receiving additional remittances improves the predicted probability of elderly adults’ SF. However, given the non-significance of remittances^2^ in model 2, we do not report the results for PF.

#### 3.2.3. Association between Other Demographic Variables, Social and Physical Functioning

The SF odds of married elderly adults decreased by 71%% (AOR = 0.286; CI = 0.243–0.336; *p* < 0.01) relative to the unmarried elderly adults. Additionally, SF odds of literate elderly adults are 12% (AOR = 0.876; CI = 0.755–1.016; *p* < 0.1), while their PF are higher by 57% (AOR = 1.570; CI = 1.412–1.745; *p* < 0.01) relative to the illiterate elderly adults. Furthermore, urban-dwelling elderly adults have SF odds that are 36% (AOR = 0.637; CI = 0.570–0.712; *p* < 0.01) lower than those of rural-dwelling elderly adults. Finally, only elderly adults residing in the southwest of Nigeria have higher SF odds (AOR = 1.437; CI = 0.959–2.166; *p* < 0.1), while only northwestern elderly adults have better PF odds (AOR = 1.638; CI = 1.049–2.559; *p* < 0.05) compared to the elderly adults in the security-risk northeast.

## 4. Discussion

As Nigeria is experiencing a boom in its population of elderly adults in a poor environment with inadequate social security, we studied the efficacy of an alternative source of income for elderly adults by (i) examining the association between remittances and healthy aging for all age groups, (ii) investigating the differences in health outcomes between remittance recipients and non-recipients, (iii) and identifying the minimum remittance amount that engenders healthy aging. We found that remittances are associated nonlinearly with healthy aging; hence, the effects of remittances on healthy aging depend on the amount received. Additionally, elderly remittance recipients had improved social functioning relative to non-recipients.

Our results show significant and nonlinear associations between remittances and healthy functioning. Remittances show an abundant significant influence on health outcomes [22,23,61]. Though the implications of this significance are mixed for our study’s two health indicators, the result establishes remittances as a credible influencer of healthy functioning in Nigeria. Since remittances are associated with reducing infant mortality and increased infertility, Zhunio et al. also obtained mixed results in their research [46]. Furthermore, given our findings and following the logic presented in the healthy aging and life-course aging theory (as discussed in the introductory section), we expected the middle-aged and the elderly to have lower healthy aging odds relative to the young population. Hence, analyzing the within-group differences in terms of outcome provides better insights in the effects associated with receiving remittances.

While those who receive remittances (young and middle-aged) have significantly higher SF probabilities than non-recipients, remittance recipients have significantly lower PF probabilities than non-recipients in all age groups. SF captured the participants’ ability to engage in income-generating social activities in this study. As poverty is rife in Nigeria [35] and since only 4% of the study participants engaged in wage-earning activities, the young and middle-aged study participants who engaged in private agricultural and non-agricultural ventures (40% and 24%, respectively) would have boosted their SF by investing these additional remittance incomes in their private income-generating social activities. In their Guatemala study, Adams and Cuecuech lent credence to this argument, observing that remittance-receiving households spend more on investments than non-recipient households [62]. Alternatively, Kan’s Tajikistan study highlighted other channels through which remittances improve SF [63]. He explained that remittances could reduce the number of work-leave days due to sudden or chronic illness. Additionally, due to its purchasing power augmentation, remittances enhance the decisions that recipients make to receive professional care, shortening their sick leave. Lastly, he expressed that remittance recipients can afford more nutritious foods that prevent sickness and that keep them engaged at work. The arguments are credible explanations for the increased SF probabilities observed in remittance recipients. Unfortunately, the social functioning probabilities of the elderly are similar between remittance recipients and non-recipients. Elderly Nigerians aged 66 years and above are the most vulnerable section of society because they are socially isolated [64] and physically and institutionally abused [65]; hence, remittances, as some researchers observed, may not adequately compensate for these challenges in the absence of a functional social security framework [47,48,49].

Conversely, the remittance recipients in our study have significantly lower PF probabilities than non-recipients. Though this result is counterintuitive since we expect additional resources to afford better health outcomes, a recent study found remittance recipients with poorer PF odds than non-recipients [18]. There are plausible explanations for this. First, non-recipients could generally have better PF. Second, remittance recipients could spend this additional income on expenditures that are not related to health or on unhealthy consumption habits that deteriorate their health [66,67]. Interestingly, the remittance spending patterns indicated in the NLSS offer probable evidence for this, as shown in Table 5 below.

Of the 21% study of participants who reported their remittance expenditure patterns, they, on average, only spent 4.7% of their remittances on medical care-related expenditures, with 81.8% being spent on consumption. Though 60–80% of the Nigeria’s population of elderly adults require regular medical attention [16], it is surprising that only 4.7% of the received remittances go to medical expenses. There are several possible reasons for this: firstly, remittances may not be large enough to sustain a living; hence, they do not provide much to spare in terms of medical expenses [18]. Next, elderly adults might deliberately spend less on medical expenses because they prefer the cheaper and widespread traditional treatments that are available in Nigeria [68,69] or downplay their health challenges because of their “unrealistic optimism” in life [70,71]. Following these lines of thought, we expect that attaining healthy physical functioning in elderly adults will be complex.

The differences in these two outcomes suggest that Nigerian elderly adults could be socially engaged, even when they are in poor physical health [16]. This contradiction makes sense given the availability of caregivers, disability aids for the elderly, and mobility equipment to improve physical functioning. Hence, it is possible to have limited social activities with poor physical health. For instance, many elderly adults cannot sit for long or lift heavy objects at work due to back pain. Additionally, blind people have social functioning capacity though they need physical assistance.

Furthermore, the AOR from our logistic regression shows that remittances and SF are nonlinearly associated, indicating that improvements in SF are probably associated with receiving higher remittance amounts. The predictive margins estimated this remittance threshold amount to be NGN 1,200,00 per annum (see Figure 3). Though several scholars studied the nonlinear relationship between remittances and other factors [50,51,52], studies on the nonlinear relationship between remittances and health outcomes are sparse. However, a recent study [18] supports our findings. In their study, left behind wives in India who received more than INR 35,000 s from their migrant husbands reported higher self-rated health than those who received lower remittance amounts.

### Policy Implications

Historically, the needs of elderly Nigerian adults have eluded the focus of government policies because not many people lived until elderly age. Unfortunately, this insufficient focus persists given current policies (N-Power, Tradermoni, and the National Development Plan 2021–2025), which target the younger generation. We infer policy implications based on the WHO’s global healthy aging strategies in the Nigerian context to address these.

Given Nigeria’s poverty rate and insufficient public resources (Nigeria’s 2022 annual national budget per capita is USD 195, compared to the US’s USD 18,000 [72,73]), the government may not be materially equipped to address short-term healthy aging goals. Hence, in the absence of enormous capital outlays, our discussions below focus on the government’s capacity to create an environment that fosters the healthy aging of the population in the interim.

Firstly, due to their significance, the governments’ efforts are imperative in eradicating or ameliorating the commercial, regulatory, consumer, and infrastructural barriers to remittance flow (See [74] for the remittance barrier analysis). This effort requires diplomatic engagement with other climes regarding a bilateral smoothening out of local and international financial transaction barriers. Given that Nigerian debit cards have a monthly spending limit of USD 100, restricting remittances from foreign experts, many countries also have similar remittance-inhibiting policies. Consequently, formal cross-border remittances are expensive and time-consuming, causing many to employ informal remittance transfer methods that hurt the economy. Hence, given the Nigerian government’s material inadequacies, the Central Bank of Nigeria (CBN) must increase the monthly transaction limits and step-up international collaborations to enable sufficient care of the elderly by migrants.

Secondly, since most working elderly adults engage in private income-generating activity, the government must channel resources to the country’s population of elderly adults to enhance their social functioning. The CBN should incentivize commercial and microfinance banks to extend cheap credit to businesses belonging to credible elderly adults since high lending rates discourage borrowing and growth. Increasing credit distribution to businesses owned by credible elderly adultscan improve their earnings and social functioning. The government may consider implementing specific programs for elderly adults that offer tax incentives, subsidized agricultural inputs, facilitated export-import transactions, and the construction of elderly-friendly infrastructure in public spaces.

Thirdly, to significantly improve the physical functioning of elderly adults, the government must consider mandatory insurance subscriptions for the elderly. Most young and old Nigerian adults do not subscribe or contribute to insurance; hence, they cannot access medical help due to financial constraints. Our study shows that remittances could not sufficiently improve the physical functioning of recipients due to inadequate social security and the poverty-prevalent environment. Therefore, a mandatory insurance policy is needed to improve the population’s access to healthcare in both the short and long term. The population would resist the policy, but the government should implement it through the National Health Insurance Scheme (NHIS).

The government must end the corruption in the social security system by ensuring a prompt pension and other social security payments to its employees and by ensuring that private businesses provide some social security benefits to their employees. The inability of the government and private companies to fulfill their social security obligations render elderly adults vulnerable to financial, emotional, and physical troubles. The provision of social security is necessary to augment remittance income, especially given the difference between the average amount of remittances and the predicted remittance amount that engenders social functioning. Hence, Nigeria’s Pension Commission (PENCOM) must adequately supervise, regulate, and prosecute defaulting officials and businesses, as empowered by the Pension Reform Act.

Finally, governments and communities must encourage a good perception of aging by reinventing the elderly adults’ appreciation and adoration days (when aging was synonymous with wisdom). A good perception of aging would reduce the anxieties that young people have about aging and positively influence society’s interaction with elderly adults. Specifically, this can be achieved by actively promoting television programs targeted at elderly adults, instituting national “elderly day” celebrations (similar to mothers, fathers, and children’s days), and routinely honoring elderly professionals nationally and in the local communities. We believe this would increase empathy towards elderly issues, increase the flow of remittances to the elderly and improve social security provisions in the long run.

The implications discussed above reflect the WHO’s four essential healthy aging requirements: creating an age-friendly environment, improving the health system, developing social security system for long-term care, and creating a positive perception of aging. We believe that addressing discussions in the Nigerian context would significantly improve the prospects for healthy aging in Nigeran elderly adults.

## 5. Conclusions

Though population aging is a global phenomenon with attendant healthy aging issues, socioeconomic challenges and insufficient private and public resources make attaining healthy aging more problematic for the Nigerian elderly adults relative to those living in other climes. Previous literature on Nigeria has focused on the perceived role of family support in improving the health of elderly adults without specific information on the quantity of this support as relevant to improving health. Hence, our study filled this gap by identifying the minimum amount of remittance support needed to enhance healthy aging in Nigeria.

While the results showed remittances to a viable, healthy aging-improving agent, we observed that this improvement is only associated with higher remittance amounts. More alarming is the difference between the average remittance amount and the predicted amount that engenders healthy aging. Given this significant difference, efforts must be intensified to attract more remittances in the short term as well as the development of an efficient social security system in Nigeria. In this regard, we suggested some policy goals based on the WHO’s healthy aging strategies in the policy recommendation section to initiate and attain both short- and long-term healthy aging for Nigerian elderly adults.

This study has several limitations, including our inability to measure our findings over time due to the unavailability of longitudinal data. Additionally, missing data for many health variables limited our analysis options. We believe that future nationally representative surveys in Nigeria would present more complete data for analysis. Lastly, more healthy aging research is required to formulate a holistic, healthy aging framework for Nigeria.

## Figures and Tables

**Table 1:** Sample Characteristics.

Variables	Total Sample Proportions	The Young Proportions	Middle-Aged Proportions	The Elderly Proportions
Physical Functioning				
Low Functioning	0.103	0.045	0.193	0.511
High Functioning	0.897	0.955	0.807	0.489
Social Functioning				
Low Functioning	0.364	0.404	0.175	0.468
High Functioning	0.636	0.596	0.825	0.532
Remittances				
No Remittances	0.721	0.778	0.621	0.352
Received Remittances	0.279	0.222	0.379	0.648
Remittance Amount				
=0	0.721	0.778	0.621	0.352
>0	0.279	0.222	0.379	0.648
Marital Status				
Married	0.517	0.431	0.837	0.450
Unmarried	0.483	0.569	0.163	0.550
Population groups				
Young	0.745	1.000		
Middle-aged	0.192		1.000	
The Elderly	0.063			1.000
Sex				
Male	0.486	0.481	0.502	0.496
Female	0.514	0.519	0.498	0.504
Literacy				
Illiterate	0.433	0.376	0.558	0.733
Literate	0.567	0.624	0.442	0.267
Residential Area				
Urban	0.296	0.297	0.297	0.710
Rural	0.704	0.703	0.703	0.290
Geopolitical Zone				
North-Central	0.205	0.211	0.187	0.195
Northeast	0.183	0.194	0.150	0.156
Northwest	0.219	0.228	0.193	0.207
Southeast	0.123	0.107	0.168	0.152
South-South	0.142	0.142	0.144	0.143
Southwest	0.128	0.118	0.158	0.147

Area total of 74,287 observation are included the total sample. The young population is represented by 55,350 responses, middle-aged is represented by 14,281 responses, and the elderly population is represented by 4656 responses.

**Table 2:** The logistic regression on Nigerians’ social and physical functioning status.

Variables	Healthy Aging Indicators			
	Social Functioning		Physical Functioning	
	AOR	CI	AOR	CI
Ref: Young population				
Middle Aged	1.985 ***	1.665–2.367	0.236 ***	0.207–0.270
Elderly	0.669 ***	0.559–0.801	0.061 ***	0.050–0.073
Received Remittances	1.523 ***	1.260–1.840	0.837 **	0.706–0.993
Effect Remittances				
Ref: Young population without Remittances				
Middle Aged Received Remittances	0.926	0.739–1.161	0.840 **	0.717–0.985
The Elderly Received Remittances	0.655 ***	0.532–0.807	0.963	0.776–1.195
Remittances	1.000 ***	1.000–1.000	1.000 *	1.000–1.000
Remittances Remittances	1.000 **	1.000–1.000	1.000	1.000–1.000
Demographic Factors				
Females	0.433 ***	0.333–0.562	1.064 *	0.991–1.142
Married	0.286 ***	0.243–0.336	0.968	0.872–1.074
Literate	0.876 *	0.755–1.016	1.570 ***	1.412–1.745
Urban	0.637 ***	0.570–0.712	1.112	0.958–1.292
Geopolitical Zones				
North-Central	1.261	0.721–2.207	0.942	0.532–1.668
Northwest	0.885	0.480–1.632	1.638 **	1.049–2.559
Southeast	1.319	0.903–1.928	0.883	0.537–1.451
South-South	1.240	0.760–2.024	1.055	0.591–1.883
Southwest	1.437 *	0.959–2.155	1.156	0.762–1.754
Constant	4.940 ***	3.315–7.362	14.982 ***	10.295–21.805
Observations	74,287		74,287	

AOR = Adjusted Odds Ratio; CI = Confidence Interval; ref = Reference group; *** *p* < 0.01, ** *p* < 0.05, * *p* < 0.1.

**Table 3:** Adjusted predicted probability of SF by remittance receipt status.

Variables	Margin	Confidence Interval
Young No Remittances	0.614	0.572–0.655
Young Received Remittances	0.708	0.667–0.749
Middle-aged No Remittances	0.759	0.706–0.813
Middle-aged Received Remittances	0.817	0.787–0.846
The Elderly No Remittances	0.515	0.464–0.567
The Elderly Received Remittances	0.515	0.462–0.567

**Table 4:** Adjusted predicted probability of PF by remittance receipt status.

Variables	Margin	Confidence Interval
Young No Remittances	0.957	0.951–0.963
Young Received Remittances	0.949	0.938–0.961
Middle-aged No Remittances	0.840	0.816–0.864
Middle-aged Received Remittances	0.787	0.751–0.823
The Elderly No Remittances	0.574	0.527–0.621
The Elderly Received Remittances	0.520	0.472–0.569

**Table 5:** Remittance spending patterns.

Spending Destinations	Spending Proportions
Maintenance for upkeep/subsidize consumption	81.8%
Mortgage fund for land, houses, and others	0.5%
Investment in shares, bonds, or others	0.6%
Development projects in the community	0.3%
Payments/donations to non-profit institutions	0.3%
Payments/donations to NGOs	0.1%
Payment of hospital bills	4.7%
Payment of school fees of household	8.2%
Purchase of agricultural inputs	2.0%
Construction of buildings	0.6%
Others	1.2%

Note: Expenditure patterns for 21% of the sample.

## Data Availability

The data utilized in this study are freely accessible from the World Bank website at: https://microdata.worldbank.org/index.php/catalog/3827/get-microdata (accessed on 21 July 2021). The data are made available by signing up for the platform.
